# Sports dentistry intricacies with season-related challenges and the role of athlete-centered outcomes

**DOI:** 10.3389/froh.2025.1531653

**Published:** 2025-02-13

**Authors:** André Júdice, João Botelho, Vanessa Machado, Luís Proença, Luciano M. A. Ferreira, Peter Fine, José João Mendes

**Affiliations:** ^1^ Egas Moniz Center for Interdisciplinary Research (CiiEM), Egas Moniz School of Health & Science, Almada, Portugal; ^2^ Eastman Dental Institute, University College London, London, United Kingdom

**Keywords:** sports dentistry, athletes, season-related challenges, ahtlete-centered outcomes, oral health

## Abstract

Sports dentistry is an emerging field focusing on the prevention, treatment, and management of oral health issues in athletes. This review critically examines the current state of sports dentistry, emphasizing the integration of oral health care into athletes' overall health management. The high prevalence of dental caries, erosion, and periodontal problems among athletes is linked to diet, habits, and increased risk of orofacial injuries. Routine oral health evaluations, particularly during the preseason, are recommended to tailor prevention strategies and address potential issues early on. The recently proposed Universal Screening Protocol for Dental Examinations in Sports is discussed, noting its potential limitations in terms of time and complexity. The review explores the challenges posed by athletes' demanding training and competition schedules, stressing the importance of incorporating oral health care into the medical team. Future research should investigate the feasibility and validity of self-reported questionnaires for screening oral health conditions, potentially simplifying the process for athletes. The review highlights the use of athlete-reported outcomes and self-reporting in sports dentistry as crucial for evaluating dental care effectiveness and monitoring long-term health outcomes. It concludes by emphasizing the need for streamlined, universally applicable protocols that fit athletes' schedules while providing comprehensive care, and the importance of further research to explore innovative screening tools and self-reported measures to transform routine care practices and reduce barriers to dental health for athletes.

## 1 Introduction

Part of the role of the sports medicine doctor is to help prepare elite athletes to reach the peak of their performance at the critical time, so that they win the event they have been preparing for. However, Sports Medicine doctors have very little knowledge about oral health and the implications of poor oral health on athletic performance (1). Sports Dentistry is a relatively new exciting branch of sports medicine which offers an increasing importance to Sports Medicine (2–4). Sports Dentistry is defined by the International Dental Federation (FDI) as an area “focused on preventing and treating oral cavity and stomatognathic system pathologies and injuries that arise from sports activities” (5). This is even more relevant based on the alarming levels of oral deterioration and oral health literacy reported among athletes over the past years (1, 6–8).

It is well documented that the significance of poor oral health extends beyond the oral cavity. The impact of oral health challenges can result in systemic diseases such as cardio-vascular disease, diabetes and pulmonary disease (9), although it is unclear whether there is true causality or just an association between periodontal disease and certain other systemic conditions. It addresses the unique dental and oral challenges that athletes face owing to their rigorous training and competition routines. A sports dentist's responsibilities encompass injury prevention, treatment of dental trauma, management of oral health issues, and performance enhancement through optimal dental and oral healthcare (10) as part of the Sports Medicine team (11). The ultimate goal of Sports Dentistry is to safeguard the oral health of athletes; however, the complexity relies on dealing with other key components of an athlete that may interfere with oral health, such as a tight training schedule with season-related constraints, consequences of intense training (12), nutrition (8, 13), association with systemic conditions (14) that athletes may have, mental health (15) or athlete/patient-centered outcomes (16).

Recently, several consensuses (3, 17) and one universal protocol (2) have been proposed for Sports Dentistry. First-of-a-kind proposals in this endeavor often encounter the complexities of navigating uncharted intellectual territories, where established guidelines and frameworks are scarce. We must also identify the end users of such key documents, in this case, clinicians. As an emerging field, there is a layer of difficulty dependent on the boundaries of the field, and for this reason, critically revisiting the available literature is mandatory to this progress.

Here, we revisit the increasing significance of Sports Dentistry and critically revise the current gaps in available clinical protocols. Additionally, we analyzed the challenges presented by the training and competition routines for the sports dentists. Finally, we discuss the potential value of athlete-centered outcomes in Sports Dentistry.

## 2 Oral health as an integrative part of athlete's overall health

Sports dentistry is an essential component in maintaining the oral health and overall well-being of athletes. Its significance transcends merely preventing dental injuries, as it also contributes to the broader health and performance of athletes. To fully leverage its potential, Sports Dentistry should be acknowledged and integrated as a crucial part of a multidisciplinary sports medicine team, collaborating with other specialists to provide comprehensive care and optimize an athlete's performance.

Previous studies have shown that athletes’ oral health is poor. Based on a systematic review, 15%–75% of athletes across all sports, presented dental caries, 35%–85% erosion, and 0%–15% periodontal problems (1, 10).

One of the reasons for the appearance of this high prevalence can be attributed to the diet and habits of athletes. Athletes regularly use sports, energy drinks, and carbohydrate-rich nutrients in drinks or gels during training and competition. At the same time, dehydration and stress caused during sports activity aggravate the effect of carbohydrates and acidic components on the dental hard tissues, reducing the protective effect of saliva (18).

Moreover, during their activity, athletes have an increased risk of injuries to the head and mouth, especially in contact sports including rugby (19), basketball (20), field hockey (21) and martial arts (22). Therefore, a key component in preventing these conditions is identifying individual risk factors, pertinent information, and guidance of athletes by the dentist.

Finally, individual attitudes, behaviors, and culture toward oral health are crucial parameters for achieving oral health. Unfortunately, studies have shown that athletes' knowledge of the value of oral health and risk assessment needs to be improved (17). At the same time, the percentage of athletes who make regular dental visits is insignificant.

Two studies have focused on preseason periods to assess periodontal status and dental caries with behaviors on football players and skiers (7, 23). In footballers, the prevalence of periodontitis was 40.9% and peri-implantitis was also observed, and some gum measures correlated with anthropometric metrics (7). Skiers had similar oral health conditions as the control group, but the percentages were high (23), which is concerning given the importance of oral health for athletes. There are few studies on the oral health status of athletes at the preseason stage, which is a crucial time for building a strong foundation to endure the entire season ahead (24). It also presents a good opportunity to restore or repair uneven health levels. Oral health is equally important and should not be overlooked during this stage but should be a part of the athletes pre-season screening (25). To avoid dental procedures that may have negative consequences for health and performance, some athletes opt to schedule dental procedures, such as wisdom tooth removal, due to their morbidity and potential implications for performance and well-being during the close season. Nevertheless, this is still a gray zone that requires further study to better clarify when and how to proceed.

**Figure 1:**
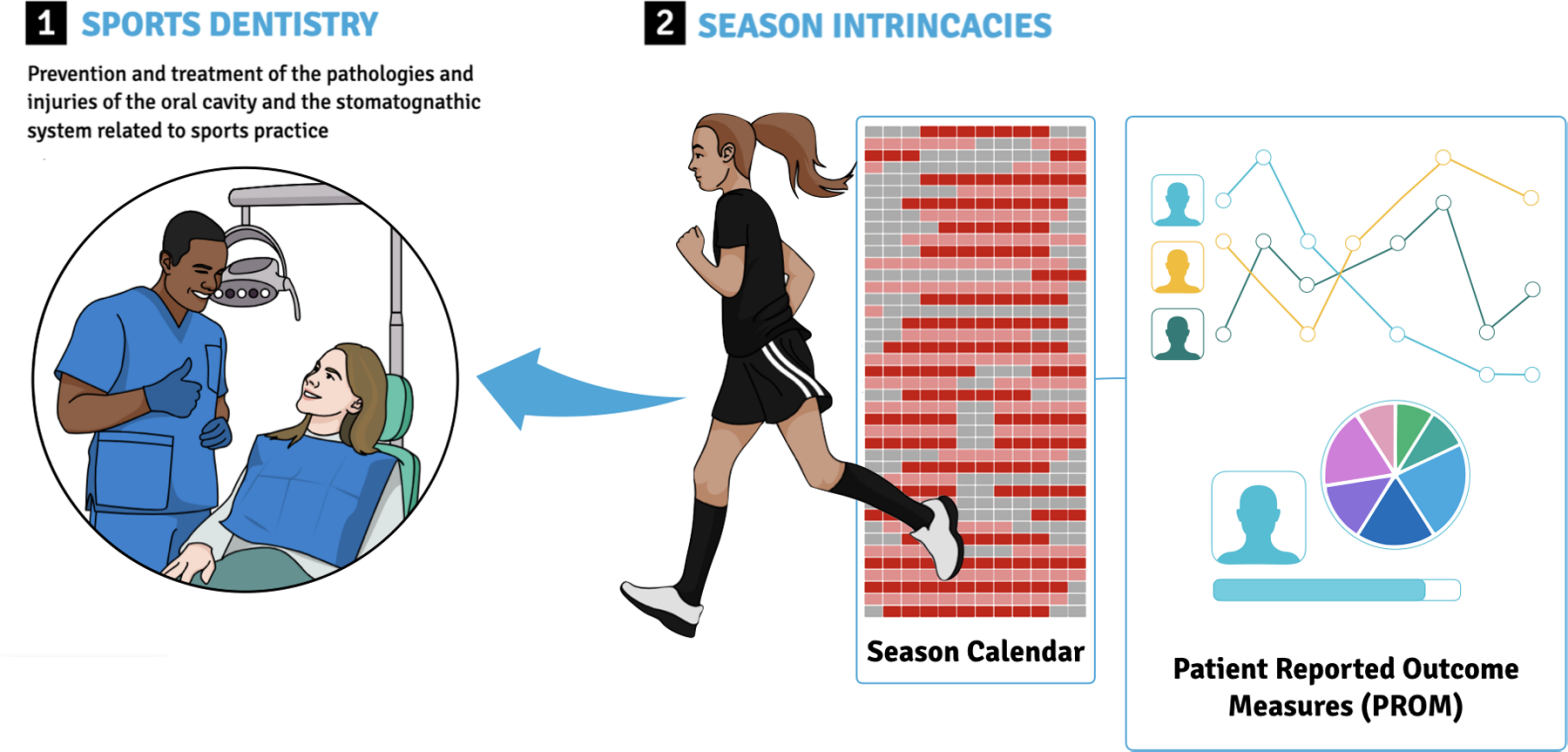
Season-related intricacies due to high variable schedule and intensity trainings and association with patient-reported outcome measures.

Fully trained dental professionals should conduct routine oral health evaluations, particularly before the season begins, to customize prevention strategies and address any issues early on (Figure 1). Leading national sports organizations and policymakers should spearhead the implementation of this approach. The International Olympic Committee, recognizing the importance of safeguarding athletes’ health, including dental well-being, has announced the establishment of a dedicated “Oral Health Department” in preparation for the 2024 Paris Olympic and Paralympic Games. International sports governing bodies promote a comprehensive strategy to ensure athletes' welfare and performance (26). Managing to integrate a multidisciplinary health team approach has been shown to promote a rigorous monitoring and management of athlete health and performance facilitating a balanced approach to training and competing decisions (27).

## 3 Current observation protocols in sports dentistry

In 2023, the European Association for Sports Dentistry (EA4SD) and the Academy for Sports Dentistry (ASD) in the USA, proposed a Universal Screening Protocol for Dental Examinations in Sports (USPDES) (2). The USPDES is an inclusive protocol, comprises six sections and results in a final color-based (defined as green as “no pathological and/or functional findings”, yellow as “presence of at least one pathological or functional findings” and red as “multiple severe pathological and/or functional findings”) categorization that will render an eligibility authorization to practice sports based on a comprehensive and mostly oral-based inspection.

The four main goals of USPDES were: (i) to be brief and quick; (ii) to allow collecting the maximum amount of data about the overall health status of the stomatognathic system as well as to provide a more efficient version of data collection; (iii) to include basic information about the oral health conditions that will benefit the athlete and team; (iv) to adopt the instructions and codification of the FDI (2).

Screening/early recognition of disease is well established in health care. Use of standard clinical indices such as the International Caries Detection and Assessment System (ICDAS) (28), the Basic Periodontal Examination (BPE) (29) and the Basic Erosive Wear Examination (BEWE) (30) encourage greater clarity when reporting prevalence of disease for epidemiologic studies and when providing athlete feedback regarding individual risk (11).

A short and concise instrument for screening is vital to its widespread implementation within the complexity of a season and the multiple areas in which a medical team is composed. However, we must highlight some characteristics of USPDES that may oppose these goals. This protocol includes full-mouth examination protocols for the examination of dental status (including dental caries, tooth wear, percussion tests, ICDAS and DMF), periodontal status, temporomandibular (TMJ) joint status and dental wear. The following diagnostic procedures may potentially compromise the “short” and “shorter and faster to fill version”, of the screening tool as the majority of these procedures require significant time and are challenging for both patients and examiners, potentially resulting in high dropout rates and measurement inaccuracies (31–35).

Future research may investigate the viability of utilizing self-reported questionnaires as a means of screening for these conditions, with the aim of simplifying the schedule of athletes, who are typically busy and must adhere to strict timetables.

## 4 Training and competition routines challenges for oral health

**Figure 2:**
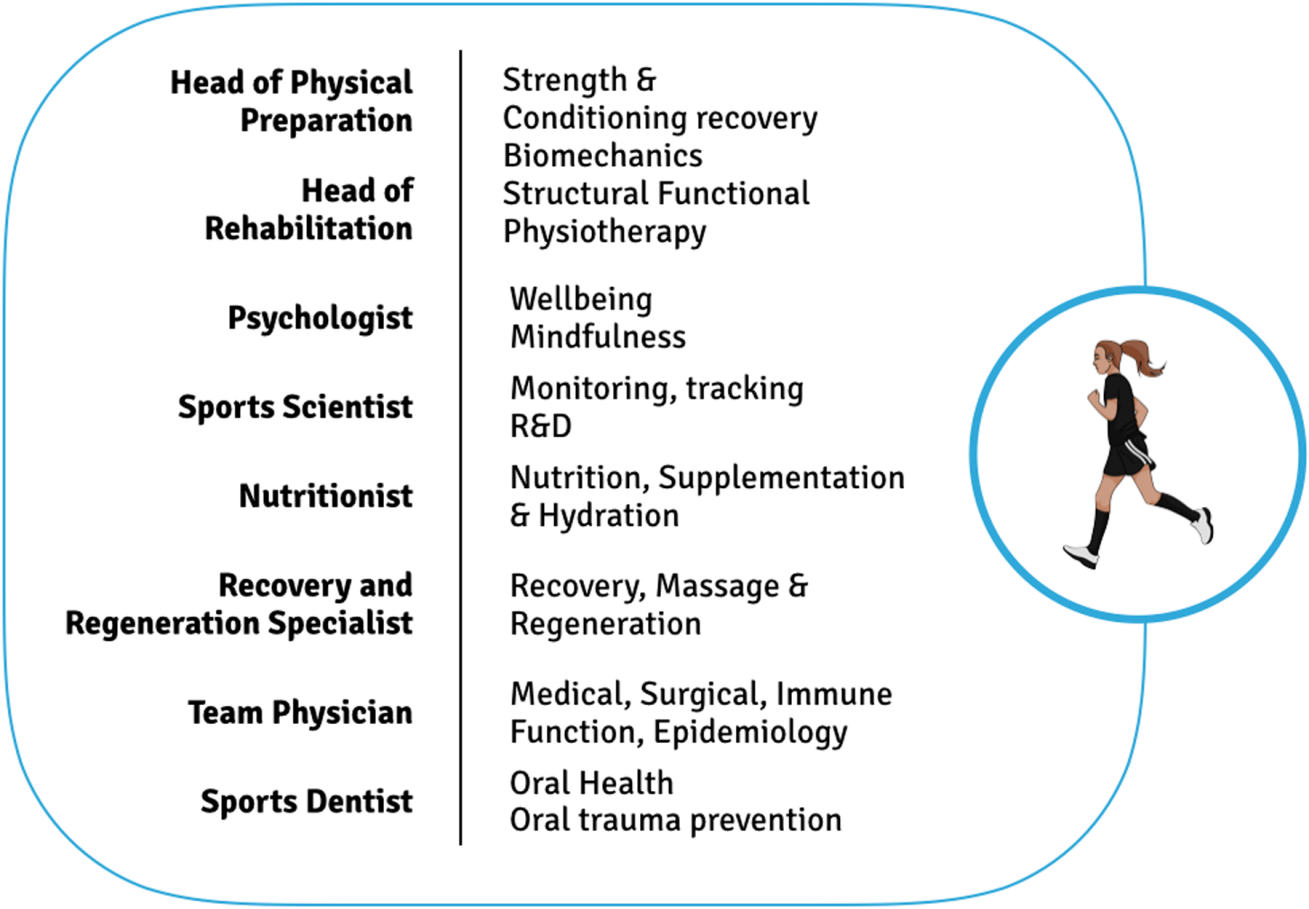
Adapted schematic of professional team sport staff including oral health specialized in sports dentistry. Modified from Calleja-González (36).

A precise routine for athletes is key for complex preparation to the competition, including mental health, physical health, technical abilities, and other aspects. In addition, elite athletes know their schedule in detail weeks in advance, as is often hard to manage unpredictable schedule needs, such as oral health care. Unless oral health care is integrated in the medical team that supports the athlete, engaging in oral health care plans is frequently a challenge both for the oral health team and the athlete (Figure 2) (36). Thus, oral health professionals are essential in safeguarding the dental well-being and overall health of athletes, addressing preventative and curative needs. Dentists contribute to athletes' optimal performance by recognizing and treating issues such as dental injuries, tooth decay, gum disease, and jaw joint disorders. Dental practitioners frequently team up with other healthcare experts, including physicians, physical therapists, and nursing staff, to ensure a comprehensive approach to athlete care. For instance, dentists may collaborate with doctors to tackle systemic conditions associated with oral health, such as infections that might provoke inflammation or hinder recovery. Working alongside nutritionists can help reduce risks associated with dietary choices, like sports beverages causing tooth erosion. These cross-disciplinary efforts promote the incorporation of oral health into broader healthcare strategies, maximizing athletes' performance.

The type of sport also seems to be linked to different levels of oral health, as elite athletes who compete in individual sports have been shown to present a worse oral situation than athletes competing in teams sports (37).

Nevertheless, the role of dentists in managing this varies across countries due to differences in healthcare systems, training, and awareness. In countries such as the United States, United Kingdom, and Australia, sports dentistry is well-established, with dental professionals collaborating closely with sports teams to provide preventive care, including custom mouthguards, dietary advice, and routine examinations. These countries often integrate oral health into athlete care plans, reflecting a high level of awareness regarding its importance. Conversely, in countries where sports dentistry is an emerging field, general dental practitioners may address oral health needs reactively, focusing on treating issues such as dental trauma or caries rather than implementing preventive measures. Access to care and awareness also exhibit variation, with disparities in the availability of specialized care and knowledge among athletes and coaches. Countries at the forefront of sports dentistry often implement best practices, such as mandatory oral health screenings for professional athletes or Olympic teams, promoting a proactive approach. However, in numerous regions, limited resources and cultural differences hinder the prioritization of oral health. To address these disparities, international collaboration, standardized guidelines, and educational initiatives could contribute to ensuring consistent oral healthcare for athletes globally, emphasizing its role in enhancing athletic performance and long-term health.

## 5 Patient-reported outcomes and self-report in sports dentistry

Patient/athlete-reported outcomes (PROs) and self-reporting in sports dentistry are critical tools that help in evaluating the effectiveness of dental care, understanding athlete satisfaction, and monitoring recovery or long-term health outcomes. These methods empower patients/athletes to provide direct feedback on their oral health, functionality, and quality of life, especially in the context of sports-related dental injuries and treatments. In a recent review, several advantages and unfounded disadvantages were discussed about self-reports (38). Overall, Corneille & Gawronski (2024) highlight that self-reports are reliable, its measures seem to be better at predicting behavior than implicit measures, and its flexibility (used to assess thoughts and feelings about complex situations).

In this regard, there is a wide and vast field of study in Sports Dentistry. Storrer et al. (2021) explored the impact of oral herpes lesion in Brazilian para-athletes oral health-relate quality of life (OHRQoL) (39), in Peruvian athletes (40), while oral health self-reports were used in a cross-sectional of biathletes and cross-country skiers (23). Also, a systematic review by Gallagher et al. (2017) studied which athlete-reported outcome measures of performance have been used to measure the impact of injury and illness on performance in sport and assess evidence to support their validity (41).

## 6 Future perspectives

As the field of Sports Dentistry continues to evolve, its integration into broader healthcare frameworks for athletes upholds enhanced outcomes in oral health, yet for athletic performance the level of evidence is still weakly robust. Thus, it is essential to develop streamlined, universally applicable protocols that accommodate the demanding schedules of elite athletes while providing comprehensive care. Further research is warranted to explore self-reported measures and innovative screening tools, potentially transforming routine care practices and reducing barriers to dental health for athletes. This progression not only aligns with holistic athlete health strategies but also boosts the role of Sports Dentistry as a fundamental component of athlete's care management, underscoring its importance in sports medicine's multidisciplinary approach.

## References

[1] Ashley P Di Iorio A Cole E Tanday A Needleman I. Oral health of elite athletes and association with performance: a systematic review. Br J Sports Med. (2015) 49:14–9. 10.1136/bjsports-2014-093617 25388551 PMC4316843

[2] Stamos A Engels-Deutsch M Cantamessa S Dartevelle J Crouzette T Haughey J A suggested universal protocol for dental examination in sports. Dent Traumatol (2023) 39:edt.12863. 10.1111/edt.12863 37367210

[3] Stamos A Mills S Malliaropoulos N Cantamessa S Dartevelle J-L Gündüz E The European Association for Sports Dentistry, Academy for Sports Dentistry, European College of Sports and Exercise Physicians consensus statement on sports dentistry integration in sports medicine. Dent Traumatol. (2020) 36:680–4. 10.1111/edt.12593 32790959

[4] Dvorak J Junge A. Twenty years of the FIFA medical assessment and research centre: from “medicine for football” to “football for health.”. Br J Sports Med. (2015) 49:561–3. 10.1136/bjsports-2015-094805 25878070 PMC4413737

[5] Sports Dentistry | FDI. Available online at: https://www.fdiworlddental.org/sports-dentistry-0 (accessed October 11, 2023).

[6] Needleman I Ashley P Meehan L Petrie A Weiler R McNally S Poor oral health including active caries in 187 UK professional male football players: clinical dental examination performed by dentists. Br J Sports Med. (2016) 50:41–4. 10.1136/bjsports-2015-094953 26527674

[7] Botelho J Vicente F Dias L Júdice A Pereira P Proença L Periodontal health, nutrition and anthropometry in professional footballers: a preliminary study. Nutrients. (2021) 13:1792. 10.3390/nu13061792 34070244 PMC8225082

[8] Khan K Qadir A Trakman G Aziz T Khattak MI Nabi G Sports and energy drink consumption, oral health problems and performance impact among elite athletes. Nutrients. (2022) 14:5089. 10.3390/nu14235089 36501119 PMC9738880

[9] Kane SF. The effects of oral health on systemic health. Gen Dent. (2017) 65:30–4. 29099363

[10] Needleman I Ashley P Fine P Haddad F Loosemore M de Medici A Oral health and elite sport performance. Br J Sports Med. (2015) 49:3–6. 10.1136/bjsports-2014-093804 25263651 PMC4316856

[11] Gallagher J Fine P Ashley P Needleman I. Developing the role of the sports dentist. Br Dent J. (2021) 231:544–6. 10.1038/s41415-021-3612-9 34773016

[12] Flockhart M Nilsson LC Tais S Ekblom B Apró W Larsen FJ. Excessive exercise training causes mitochondrial functional impairment and decreases glucose tolerance in healthy volunteers. Cell Metab. (2021) 33:957–970.e6. 10.1016/j.cmet.2021.02.017 33740420

[13] Needleman I Ashley P Fairbrother T Fine P Gallagher J Kings D Nutrition and oral health in sport: time for action. Br J Sports Med. (2018) 52:1483–4. 10.1136/bjsports-2017-098919 29853456

[14] Botelho J Mascarenhas P Viana J Proença L Orlandi M Leira Y An umbrella review of the evidence linking oral health and systemic noncommunicable diseases. Nat Commun. (2022) 13:7614. 10.1038/s41467-022-35337-8 36494387 PMC9734115

[15] MacIntyre TE Jones M Brewer BW Van Raalte J O’Shea D McCarthy PJ. Editorial: mental health challenges in elite sport: balancing risk with reward. Front Psychol. (2017) 8:1892. 10.3389/fpsyg.2017.01892 29118734 PMC5661081

[16] Nascimento GG Raittio E Machado V Leite FRM Botelho J. Advancing universal oral health coverage via person-centred outcomes. Int Dent J. (2023) 73: S0020653923000977. 10.1016/j.identj.2023.06.006 37684172 PMC10658430

[17] Needleman I Ashley P Fine P Haddad F Loosemore M de Medici A Consensus statement: oral health and elite sport performance. Br Dent J. (2014) 217:587–90. 10.1038/sj.bdj.2014.1000 25415018

[18] Ntovas P Loumprinis N Maniatakos P Margaritidi L Rahiotis C. The effects of physical exercise on Saliva composition: a comprehensive review. Dent J (Basel). (2022) 10:7. 10.3390/dj10010007 35049605 PMC8775020

[19] Padilha ACL Constante HM Fronza HP Coto NP. Orofacial trauma and mouthguard use in Brazilian rugby union players. Dent Traumatol. (2021) 37:53–7. 10.1111/edt.12592 32794620

[20] Azodo CC Odai CD Osazuwa-Peters N Obuekwe ON. A survey of orofacial injuries among basketball players. Int Dent J. (2011) 61:43–6. 10.1111/j.1875-595X.2011.00009.x 21382033 PMC9374803

[21] Hendrick K Farrelly P Jagger R. Oro-facial injuries and mouthguard use in elite female field hockey players. Dent Traumatol. (2008) 24:189–92. 10.1111/j.1600-9657.2007.00527.x 18352922

[22] Bruggesser S Kühl S Solakoglu Ö Filippi A. The prevalence of orofacial injuries in judo: a cross-sectional study. Dent Traumatol. (2020) 36:411–6. 10.1111/edt.12547 31994310

[23] Merle CL Rott T Challakh N Schmalz G Kottmann T Kastner T Clinical findings and self-reported oral health status of biathletes and cross-country skiers in the preseason - a cohort study with a control group. Res Sports Med. (2022) 32:1–15. 10.1080/15438627.2022.2090251 35762035

[24] The Importance of Preseason. SportsPerformanceTracking-USA. Available online at: https://us.sportsperformancetracking.com/blogs/spt-playbook/the-importance-of-preseason (accessed October 12, 2023).

[25] Meehan L. Screening for dental disease amongst elite athletes. In: Fine PD Louca C Leung A, editors. Sports Dentistry. Wiley (2018). p. 159–89. 10.1002/9781119332619.ch10

[26] Matheson GO Klügl M Engebretsen L Bendiksen F Blair SN Börjesson M Prevention and management of non-communicable disease: the IOC consensus statement, Lausanne 2013. Br J Sports Med. (2013) 47:1003–11. 10.1136/bjsports-2013-093034 24115479

[27] Dijkstra HP Pollock N Chakraverty R Alonso JM. Managing the health of the elite athlete: a new integrated performance health management and coaching model. Br J Sports Med. (2014) 48:523–31. 10.1136/bjsports-2013-093222 24620040 PMC3963533

[28] Ismail AI Sohn W Tellez M Amaya A Sen A Hasson H The international caries detection and assessment system (ICDAS): an integrated system for measuring dental caries. Community Dent Oral Epidemiol. (2007) 35:170–8. 10.1111/j.1600-0528.2007.00347.x 17518963

[29] British Society of Periodontology. Basic Periodontal Examination (BPE) (2019)

[30] Bartlett D Ganss C Lussi A. Basic erosive wear examination (BEWE): a new scoring system for scientific and clinical needs. Clin Oral Invest. (2008) 12:65–8. 10.1007/s00784-007-0181-5 PMC2238785 18228057

[31] Owens JD Dowsett SA Eckert GJ Zero DT Kowolik MJ. Partial-mouth assessment of periodontal disease in an adult population of the United States. J Periodontol. (2003) 74:1206–13. 10.1902/jop.2003.74.8.1206 14514235

[32] Kingman A Susin C Albandar JM. Effect of partial recording protocols on severity estimates of periodontal disease. J Clin Periodontol. (2008) 35:659–67. 10.1111/j.1600-051X.2008.01243.x 18513337

[33] Castro ALS Vianna MIP Mendes CMC. Comparison of caries lesion detection methods in epidemiological surveys: CAST, ICDAS and DMF. BMC Oral Health. (2018) 18:122. 10.1186/s12903-018-0583-6 29980199 PMC6035475

[34] Frencken JE Giacaman RA Leal SC. An assessment of three contemporary dental caries epidemiological instruments: a critical review. Br Dent J. (2020) 228:25–31. 10.1038/s41415-019-1081-1 31925370

[35] Campus G Cocco F Ottolenghi L Cagetti MG. Comparison of ICDAS, CAST, Nyvad’s criteria, and WHO-DMFT for caries detection in a sample of Italian schoolchildren. IJERPH. (2019) 16:4120. 10.3390/ijerph16214120 31731559 PMC6862073

[36] Calleja-González J Bird S Huyghe T Jukic I Cuzzolin F Cos F The recovery umbrella in the world of elite sport: do not forget the coaching and performance staff. Sports. (2021) 9:169. 10.3390/sports9120169 34941807 PMC8705456

[37] De La Parte A Monticelli F Toro-Román V Pradas F. Differences in oral health Status in elite athletes according to sport modalities. Sustainability. (2021) 13:7282. 10.3390/su13137282

[38] Corneille O Gawronski B. Self-reports are better measurement instruments than implicit measures. Nat Rev Psychol. (2024) 3:835–46. 10.1038/s44159-024-00376-z

[39] Storrer CLM Costa EE Scariot R Pivetta Petinati MF Winckler C Deliberador TM Bruxism and type of breathing as factors associated with oral herpes lesion in Brazilian para-athletes. Spec Care Dentist. (2021) 41:700–6. 10.1111/scd.12616 34131935

[40] Márquez-Hidalgo J Zamora-Campos D Acurio-Benavente P Kinoshita-Rivas H López-Rodriguez G Moreno-Sekula K Relationship between the quality of life and oral health in athletes at a Peruvian university. Gen Dent. (2020) 68:73–7. 32857054

[41] Gallagher J Needleman I Ashley P Sanchez RG Lumsden R. Self-reported outcome measures of the impact of injury and illness on athlete performance: a systematic review. Sports Med. (2017) 47:1335–48. 10.1007/s40279-016-0651-5 27995537 PMC5488135

